# Associations of Local Comprehensive Smoke-Free Ordinances With Cancer Incidence Trends in Louisiana

**DOI:** 10.5888/pcd23.250336

**Published:** 2026-07-23

**Authors:** Taylor C. Johnson, Malesa Pereira, Earl Nupsius Benjamin-Robinson, Donna Williams

**Affiliations:** 1 Louisiana Cancer Prevention and Control Programs, School of Public Health, Louisiana State University Health Sciences Center–New Orleans, New Orleans, Louisiana; 2 Louisiana Cancer Research Center, New Orleans, Louisiana

## Abstract

**Introduction:**

Louisiana has a high prevalence of smoking-related cancers. Although the 2007 Louisiana Smoke-Free Air Act restricted smoking in many public places, exemptions for bars, casinos, and other venues left many workers unprotected. Comprehensive local ordinances were later adopted in Orleans Parish (2015) and Ouachita Parish (2017).

**Methods:**

We conducted an ecological analysis of Louisiana Tumor Registry data from 2007 through 2022 to examine age-adjusted incidence of lung cancer and tobacco-related cancers. Orleans and Ouachita Parishes were compared with parishes covered only by the partial statewide law. Trends were assessed with Joinpoint regression using annual percent change (APC), average annual percent change (AAPC), and tests of parallelism.

**Results:**

In the primary parish-level analysis, lung cancer trends differed between ordinance and nonordinance parishes (*P* = .04). Orleans Parish declined steadily (APC, −3.02%; *P* < .001). Ouachita Parish showed a 2016 joinpoint, preceding implementation of the 2017 smoke-free ordinance, followed by a post-2016 decline of −7.33% annually (*P* = .01). In sensitivity analyses combining Orleans and Ouachita Parishes, lung cancer declined more rapidly than in the remainder of Louisiana (AAPC, −3.08% vs −1.70%; *P* = .004); tobacco-related cancers also declined more rapidly (−1.26% vs −0.64%; *P* = .02).

**Conclusion:**

Comprehensive local smoke-free ordinances were associated with more favorable long-term cancer incidence trends than Louisiana’s partial statewide law. Broader statewide coverage may help reduce tobacco-related cancer burden and inequities over time.

**Summary**

**What is already known on this topic?**

Comprehensive smoke-free ordinances reduce secondhand smoke exposure and are associated with lower prevalence and fewer tobacco-related hospitalizations.

**What is added by this report?**

In Louisiana, lung cancer incidence declined more rapidly in Orleans Parish and, after a 2016 joinpoint, in Ouachita Parish than in parishes covered only by the partial statewide law.

**What are the implications for public health practice?**

Statewide comprehensive smoke-free coverage could extend workplace protections and may help reduce long-term tobacco-related cancer burden.

## Introduction

Tobacco use remains the leading preventable cause of death in the US and accounts for approximately 1 in 3 cancer deaths; cigarette smoking is linked to 80% to 90% of lung cancer deaths (1,2). Louisiana has a high prevalence of lung cancer and smoking-attributable cancer deaths, particularly for lung, laryngeal, and oral cavity cancers (3). These statewide patterns mask substantial heterogeneity in tobacco exposure and cancer outcomes driven by structural conditions that shape living environments, workplace settings, and access to preventive health care (4–6). Secondhand smoke (SHS) exposure is disproportionately concentrated among service and hospitality workers, who are more likely to be low income and from racial and ethnic minority populations (7,8). These occupational inequities intersect with rural disadvantages. In Louisiana, rural communities, particularly those with higher proportions of Black residents, experience higher lung cancer burden associated with higher smoking prevalence, reduced access to care, and later-stage diagnosis, contributing to persistent racial disparities in incidence and survival (3,5,6).

On January 1, 2007, the Louisiana Smoke-Free Air Act prohibited smoking in most enclosed workplaces and public places but exempted stand-alone bars, casinos, private clubs, and certain entertainment venues (9,10). Smoke-free air laws reduce smoking prevalence, increase cessation, and rapidly lower cardiovascular morbidity rates (11–14); however, Louisiana’s exemptions limited population-level effects and left many hospitality workers unprotected. In response, New Orleans enacted a comprehensive smoke-free ordinance in 2015, followed by parish-wide coverage in Ouachita Parish through ordinances in the cities of Monroe and West Monroe in 2017, extending protections to previously exempt venues (9,10). Evidence from other settings suggests that stronger smoke-free ordinances are also associated with more favorable long-term lung cancer incidence trends (2,15,16). Few studies have evaluated long-term cancer outcomes associated with local smoke-free ordinances in southern states.

The primary objective of this study was to assess whether comprehensive smoke-free ordinances in Orleans and Ouachita parishes were associated with steeper declines in lung and tobacco-related cancer incidence compared with parishes covered only by Louisiana’s partial 2007 statewide law. A secondary objective was to describe race-stratified trends among non-Hispanic Black and non-Hispanic White residents.

## Methods

### Study design and data source

We conducted an ecological, population-based, longitudinal trend analysis by using incident cancer data from the Louisiana Tumor Registry (LTR), a statewide surveillance system affiliated with the Surveillance, Epidemiology, and End Results Program (SEER) (17,18). Annual age-adjusted incidence rates per 100,000 population were standardized to the 2000 US standard population. The 2007–2022 study period enabled evaluation of trends before and after implementation of local comprehensive smoke-free ordinances.

### Study population and setting

The study population included all incident cancer cases diagnosed among Louisiana residents during the study period. Tobacco-related cancers were defined according to LTR site groupings and included cancers of the lung and bronchus, oral cavity and pharynx, larynx, esophagus, stomach, colon and rectum, liver, pancreas, kidney and renal pelvis, urinary bladder, cervix, and acute myeloid leukemia (19).

### Exposure classification

Parishes were classified by smoke-free policy coverage. The intervention group included Orleans Parish, which enacted a comprehensive parish-wide smoke-free ordinance in April 2015, and Ouachita Parish, where comprehensive coverage was achieved through municipal ordinances in Monroe (April 2017) and West Monroe (May 2017). These ordinances extended 100% smoke-free protections to all indoor workplaces and public venues, including bars, restaurants, casinos, hotels, and performance spaces. All other Louisiana parishes comprised the comparison group and remained covered only by the partial 2007 Louisiana Smoke-Free Air Act. A sensitivity analysis compared combined trends in Orleans and Ouachita parishes with trends in the remaining parishes statewide.

### Covariates

Race and ethnicity were classified using the SEER race and origin recode variable, which categorizes cases as Hispanic, non-Hispanic White, non-Hispanic Black, non-Hispanic Asian, or non-Hispanic Other (20). Race-stratified Joinpoint analyses focused on non-Hispanic Black and non-Hispanic White residents because annual parish-level rates were most stable in these groups.

### Statistical analysis

Temporal trends were analyzed by using the SEER Joinpoint Regression Program (version 5.4.0.0; National Cancer Institute). Joinpoint regression identifies statistically significant inflection points in longitudinal data and estimates annual percent change (APC) and average annual percent change (AAPC) with corresponding 95% CIs, supporting evaluation of changes in population-based cancer incidence over time.

Pairwise comparison tests assessed whether cancer incidence trends differed between ordinance and nonordinance parishes, using tests of parallelism to compare trend slopes. All tests were 2-sided, with *P* < .05 considered significant.

The restricted postordinance series (2015–2022) illustrated short-term changes following enactment. The full 2007–2022 series provided the primary parish-level comparison and contextualized local ordinances within the broader statewide policy environment. Formal tests of parallelism were conducted for the full series. In sensitivity analyses, Orleans and Ouachita parishes were combined and compared with the remainder of Louisiana.

### Ethical review

Because the analysis included all incident cancer cases reported statewide and relied on deidentified, population-based surveillance data, participation rates and sample size calculations were not applicable. The study was reviewed by Louisiana State University Health Sciences Center–New Orleans and determined to be exempt from institutional review board oversight in accordance with institutional policy.

## Results

### Lung cancer incidence trends, 2015–2022

**Figure 1:**
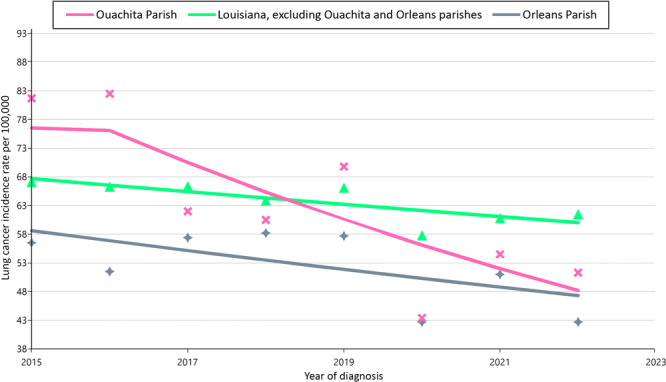
Annual age-adjusted lung cancer incidence per 100,000 population for Orleans Parish, Ouachita Parish, and the remainder of Louisiana excluding those 2 parishes, 2015–2022. Rates were age-adjusted to the 2000 US standard population. Analyses included all diagnosed lung cancer cases (ICD-O-3 codes for lung and bronchus) reported to the Louisiana Tumor Registry between 2015 and 2022 (N = statewide incident cases during that period; registry data include all Louisiana residents). Points show observed annual rates from 2015 to 2022. To preserve comparability with the primary 2007–2022 Joinpoint analysis, the fitted lines shown are the corresponding model segments from the full series; thus, the Ouachita Parish line retains the 2016 joinpoint shown in Figure 2. Joinpoint regression identified no joinpoints for any group. Abbreviation: ICD-O-3, International Classification of Diseases for Oncology, 3rd edition.

From 2015 to 2022, lung cancer incidence declined statewide, with marked variation across parishes (Figure 1). Orleans Parish began the period just under 58 cases per 100,000 population and declined steadily to approximately 46 cases by 2022 (APC, −3.32%). Ouachita Parish had the highest baseline incidence, approximately 82 cases per 100,000 in 2015, and the steepest decline, falling to about 50 cases by 2022 (APC, −7.18%). The remainder of Louisiana declined more modestly, from roughly 67 to 59 cases per 100,000 (APC, −1.65%). These postordinance patterns were descriptive and were not used for the formal parish-level hypothesis tests.

### Lung cancer incidence trends, 2007–2022

**Figure 2:**
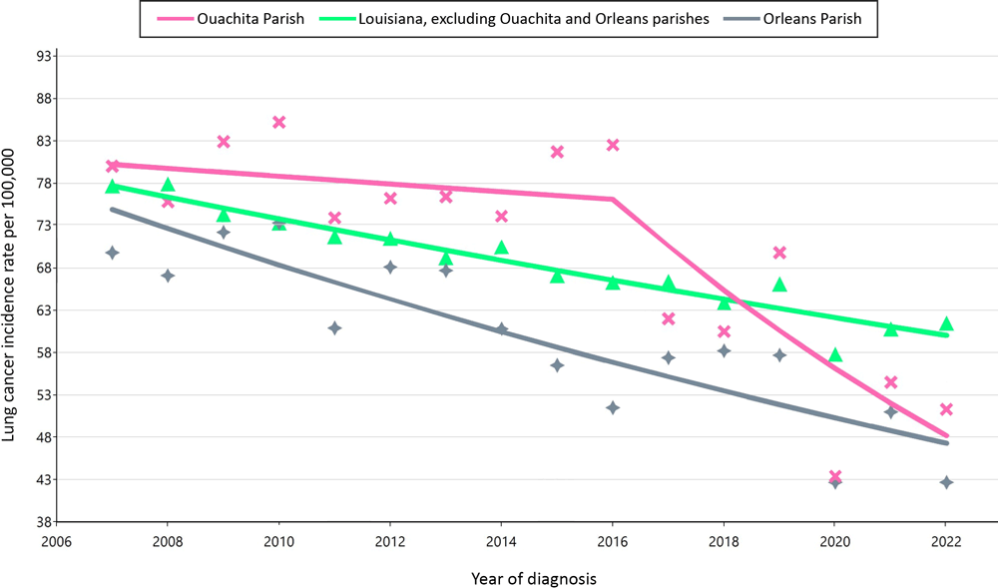
Annual age-adjusted lung cancer incidence per 100,000 population for Orleans Parish, Ouachita Parish, and the remainder of Louisiana excluding those 2 parishes, 2007–2022. Rates were age-adjusted to the 2000 US standard population. Data include all lung cancer cases (ICD-O-3 codes for lung and bronchus) reported to the Louisiana Tumor Registry from 2007 to 2022. Joinpoint regression identified no joinpoints for Orleans Parish or the rest of Louisiana. In contrast, one joinpoint was detected for Ouachita Parish: from 2007 to 2016, incidence declined slowly, followed by a sharp, significant decline from 2016 to 2022. Abbreviation: ICD-O-3, International Classification of Diseases for Oncology, 3rd edition.

From 2007 to 2022, lung cancer incidence declined in all regions, although the magnitude and timing differed (Figure 2). The remainder of Louisiana showed a consistent annual decline of −1.70% (*P* < .001) with no joinpoints. Orleans Parish experienced a steeper decline of −3.02% per year (*P* < .001), also with no joinpoints. In contrast, Ouachita Parish exhibited a joinpoint in 2016. From 2007 to 2016, incidence declined slowly and nonsignificantly (APC, −0.59%; *P* = .63), followed by a sharp and significant decline from 2016 to 2022 (APC, −7.33%; *P* = .01). Over the entire 2007–2022 period, Ouachita Parish’s average annual decline was −3.34% (*P* = .005). In the primary parish-level comparison, lung cancer trends differed between ordinance and nonordinance parishes (*P* = .04).

### Sensitivity analysis

When Orleans and Ouachita Parishes were combined and compared with the remainder of Louisiana, lung cancer incidence declined more rapidly in ordinance parishes (AAPC, −3.08% vs −1.70%; *P* = .004). For tobacco-related cancers, the combined AAPC was −1.26% versus −0.64% (*P* = .02). These findings were consistent with the parish-specific analyses.

### Tobacco-related cancer incidence trends, 2007–2022

**Figure 3:**
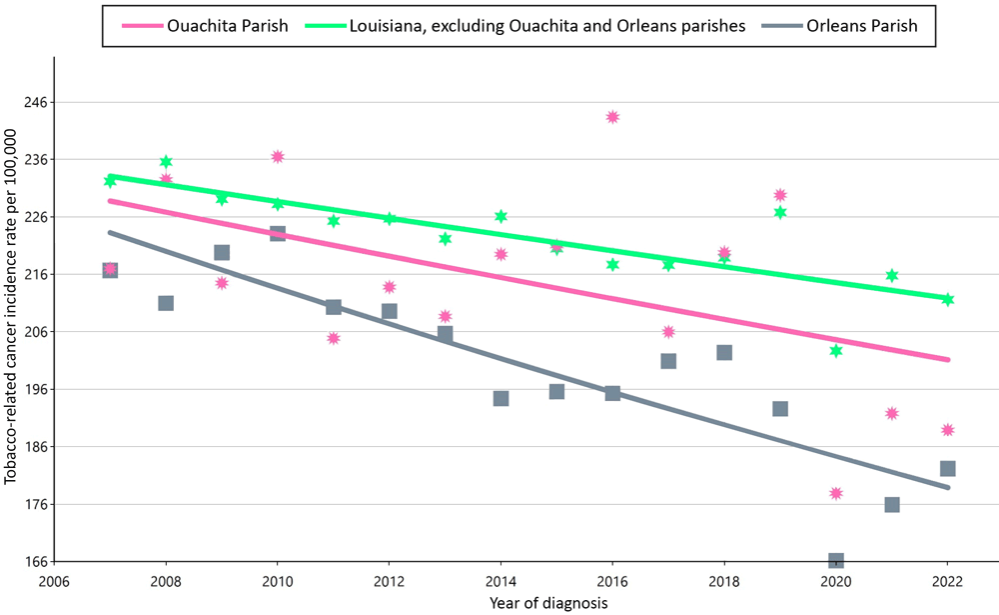
Annual age-adjusted incidence per 100,000 population for cancers causally associated with tobacco use (including cancers of the lung and bronchus, larynx, oral cavity and pharynx, esophagus, bladder, kidney, pancreas, and stomach) for Orleans Parish, Ouachita Parish, and the remainder of Louisiana excluding those 2 parishes, 2007–2022. Rates were age-adjusted to the 2000 US standard population. Data included all relevant cancer cases reported to the Louisiana Tumor Registry from 2007 to 2022. Joinpoint regression identified no joinpoints for any group.

Tobacco-related cancer incidence also declined across all regions from 2007 to 2022, though rates of decline varied (Figure 3). The remainder of Louisiana experienced the slowest reduction (APC, −0.64%; *P* < .001). Ouachita Parish showed a slightly steeper decline (APC, −0.85%; *P* = .003), whereas Orleans Parish demonstrated the most pronounced decrease (APC, −1.47%; *P* < .001). Tests of parallelism confirmed that trends differed significantly across regions (*P* = .02). 

### Race-stratified trends

**Figure 4:**
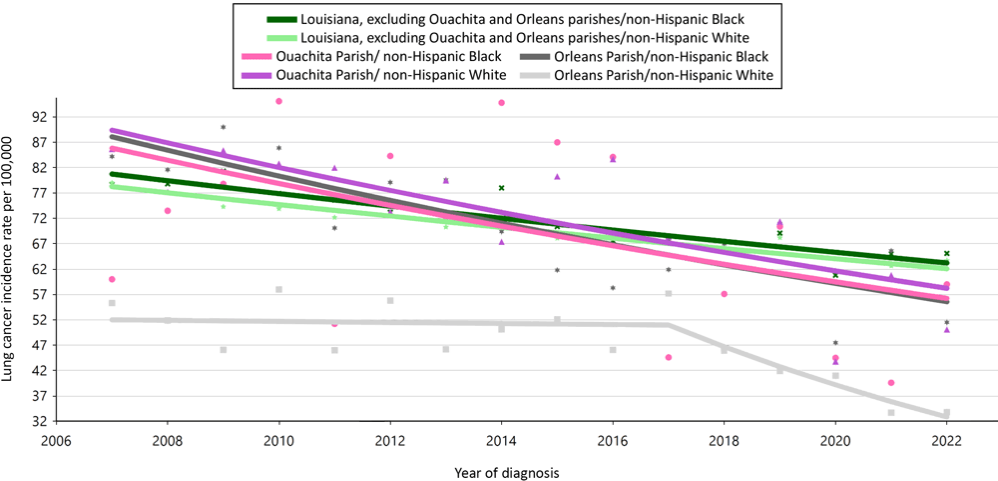
Annual age-adjusted lung cancer incidence per 100,000 population stratified by race among non-Hispanic Black and non-Hispanic White people for Orleans Parish, Ouachita Parish, and the remainder of Louisiana excluding those 2 parishes, 2007–2022. In Orleans Parish, APC = −3.03 for non-Hispanic Black; APC = −0.20 for 2007–2017 and −8.41 for 2017–2022 for non-Hispanic White. In Ouachita Parish APC = −2.78 for non-Hispanic Black and −2.82 for non-Hispanic White. In the rest of Louisiana excluding those 2 parishes, APC = −1.62 for non-Hispanic Black and −1.53 for non-Hispanic White. Rates were age-adjusted to the 2000 US standard population. Data include all lung cancer cases (ICD-O-3 codes for lung and bronchus) reported to the Louisiana Tumor Registry between 2007 and 2022. Abbreviations: APC, annual percent change; ICD-O-3, International Classification of Diseases for Oncology, 3rd edition.

**Figure 5:**
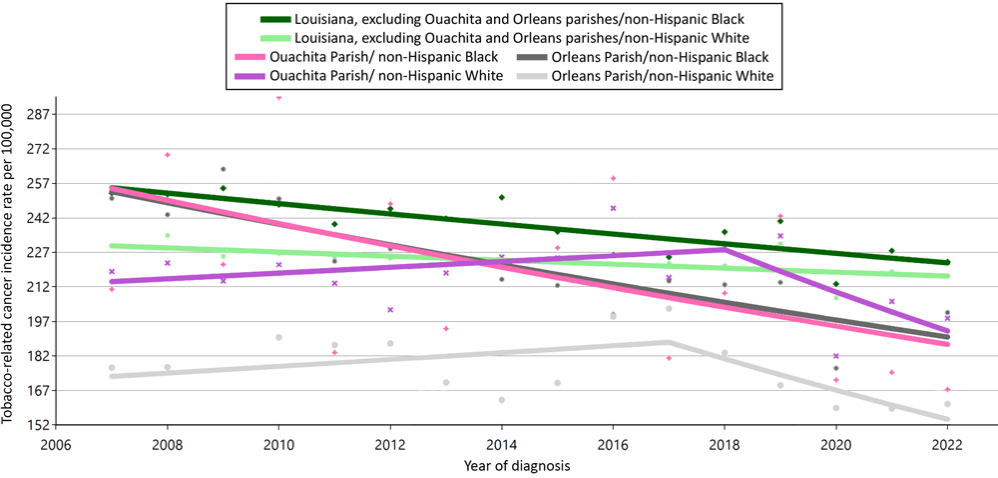
Annual age-adjusted incidence rates per 100,000 population for cancers causally associated with tobacco use (including cancers of the lung and bronchus, larynx, oral cavity and pharynx, esophagus, bladder, kidney, pancreas, and stomach) among non-Hispanic Black and non-Hispanic White people for Orleans Parish, Ouachita Parish, and the remainder of Louisiana excluding those 2 parishes, 2007–2022. Rates were age-adjusted to the 2000 US standard population. Data include all relevant cancer cases reported to the Louisiana Tumor Registry between 2007 and 2022.

Race-stratified analyses showed persistent disparities but overall declining trends for both lung cancer and tobacco-related cancers (Figure 4 and Figure 5). In Orleans Parish, lung cancer incidence declined significantly among both non-Hispanic Black and non-Hispanic White residents. Although no joinpoint was detected in the overall trend for lung cancer in Orleans Parish, race-stratified analysis identified a joinpoint for non-Hispanic White residents with a sharper post-2017 decline. In Ouachita Parish, declines were observed for both groups, although reductions among non-Hispanic Black residents were smaller and not consistently significant. For tobacco-related cancers, Orleans Parish showed significant declines among non-Hispanic Black residents and a joinpoint-driven decline among non-Hispanic White residents after 2017. In the remainder of Louisiana, declines were slower but significant for both racial groups. Pairwise comparisons indicated significant differences in slopes between ordinance and nonordinance areas for several race-specific trends, consistent with overall findings. In Orleans Parish, APC = −1.89 for non-Hispanic Black and 0.83 (2007–2017) and −3.84 (2017–2022) for non-Hispanic White. In Ouachita Parish, APC = −2.04 for non-Hispanic Black and 0.57 (2007–2018) and −4.12 (2018–2022) for non-Hispanic White. In the rest of Louisiana excluding those 2 parishes, APC = −0.91 for non-Hispanic Black and −0.39 for non-Hispanic White.

## Discussion

After comparing Orleans and Ouachita parishes, which later adopted comprehensive smoke-free ordinances, with parishes that remained under partial statewide protections, our results suggest that stronger policy coverage was associated with faster declines in cancer incidence. This study is among the first to evaluate parish-level associations between comprehensive smoke-free ordinances and long-term trends in lung and tobacco-related cancer incidence in Louisiana using population-based cancer registry data. Although Louisiana enacted statewide smoking restrictions in 2007, exemptions for bars, casinos, and other hospitality venues left many workers and patrons unprotected from SHS exposure. These gaps disproportionately affected low-income Black and Hispanic service-sector workers and reinforced inequities in tobacco exposure and tobacco-related cancer burden (4,8,21).

From 2007 to 2022, lung cancer incidence declined statewide, but the magnitude and timing of reductions varied substantially by parish. Orleans Parish experienced a steady annual decline exceeding 3%, nearly double the statewide rate, while Ouachita Parish showed relatively stable incidence through 2016, followed by a sharp decline after implementation of comprehensive ordinances in Monroe and West Monroe in 2017. The joinpoint identified in 2016 likely reflects a transitional period rather than a discrete policy effect, consistent with prior evidence that behavioral changes, voluntary business adoption, and increased public awareness often precede formal enforcement of smoke-free laws (11,15,22). Concurrent tobacco control efforts, including taxation and cessation initiatives, may also have contributed (12,23).

The long latency period of lung cancer suggests that observed declines reflect cumulative reductions in smoking prevalence and SHS exposure rather than immediate effects of policy enactment (14,16,21,24). Changes in health care access, diagnostic practices, and utilization over time also may have influenced observed incidence patterns (19). Differences in baseline exposure, smoking prevalence, socioeconomic conditions, and prior policy context further help explain divergent trajectories across parishes (3,5,6). In New Orleans, early public attention generated by the 2007 Smoke-Free Air Act, particularly its impact on restaurants and major venues, likely contributed to a gradual and sustained reduction in tobacco exposure (10). As a large urban center, Orleans Parish also has greater health care density, including specialty care, cancer screening infrastructure, and broader availability of smoking cessation resources, which may facilitate earlier diagnosis and more rapid population-level risk reduction (3). In contrast, Ouachita Parish includes more rural surrounding areas, where higher smoking prevalence, lower socioeconomic status, and more limited access to preventive and cessation services are common and may have contributed to higher baseline lung cancer incidence and slower early declines, followed by a more abrupt reduction after comprehensive smoke-free ordinances were implemented in 2017 (5,6,9).

Declines were also observed across a broader group of tobacco-related cancers. From 2007 to 2022, combined incidence rates in Orleans and Ouachita parishes declined approximately twice as fast as those in nonordinance parishes (AAPC, −1.26% vs −0.64%). Orleans Parish demonstrated the steepest reduction, whereas declines in Ouachita Parish were more modest and not statistically distinct from the rest of Louisiana. These patterns align with the heterogeneous etiology of tobacco-related cancers, many of which have long latency periods and multifactorial risk profiles that may delay detectable population-level effects of policy interventions (14,21). Long-term surveillance is therefore essential to fully capture the downstream effects of comprehensive smoke-free laws (16,24).

Race-stratified analyses highlight both persistent disparities and differential trends following implementation of comprehensive smoke-free ordinances. In Orleans Parish, lung cancer incidence declined significantly among both non-Hispanic Black and non-Hispanic White residents, although declines among Black residents were smaller, reflecting long-standing inequities in tobacco-related cancer burden (5,6,21). In Ouachita Parish, reductions among non-Hispanic Black residents were less consistent and often not significant, whereas declines among White residents were more pronounced. The differences observed between pooled and race-stratified findings suggest that important subgroup-specific trends may be partially masked when populations are combined. Race-stratified analyses identified inflection points and periods of accelerated decline that were not evident in the overall parish-level trends, highlighting the value of disaggregated approaches for understanding how tobacco control policies may differentially influence cancer outcomes across population groups. 

These findings align with prior evidence showing that Black populations experience higher cumulative exposure to SHS, lower cessation success, and disproportionate industry-driven targeting of menthol cigarette marketing through price promotions, point-of-sale placement, and tailored advertising (25). Structural barriers to health care access and cessation services further compound these inequities, particularly in rural settings (5,6). By eliminating exemptions for bars and casinos, comprehensive smoke-free ordinances extend protections to workers and communities historically excluded from smoke-free coverage and may contribute to narrowing racial disparities in tobacco-related cancer outcomes (4,7,25).

Smoking cessation remains among the most effective strategies for reducing lung cancer risk and improving cancer outcomes (14,21,26). The 2020 Surgeon General’s Report on Smoking Cessation concludes that lung cancer risk among former smokers declines substantially with time since quitting, reaching approximately half the risk of continuing smokers within 10 to 15 years, with continued declines thereafter, and documents reduced risk for multiple other tobacco-related cancers across the lifespan (14). Consistent with this evidence, smoke-free policies have been associated with rapid reductions in rates of cardiovascular morbidity, including fewer hospital admissions for myocardial infarction and stroke, reflecting early health gains following decreases in active smoking and SHS exposure (27,28). Among people already diagnosed with cancer, smoking cessation is associated with improved treatment response, fewer complications, lower risk of recurrence, and improved survival, underscoring its importance across the cancer care continuum (14,26).

By limiting exposure to SHS in indoor workplaces and public venues, smoke-free policies also shape smoking behavior at the population level. Smoke-free environments have been consistently linked to increased quit attempts, lower smoking prevalence, and greater use of cessation resources (11,13,15,29). Restricting where smoking is permitted reduces exposure to smoking cues, disrupts established routines, and reinforces tobacco-free norms in ways that support both quit attempts and sustained abstinence (15). Communities with comprehensive smoke-free coverage report higher cessation rates and stronger motivation to quit, illustrating how policy environments can influence behavior beyond individual-level interventions (11,13,29). Together, these mechanisms highlight the role of comprehensive smoke-free ordinances as structural interventions that reduce both active smoking and involuntary tobacco smoke exposure while complementing clinical and community-based tobacco control strategies.

Consistent with US and international evidence, comprehensive smoke-free policies have not been shown to harm restaurant or bar revenues and are associated with downstream economic benefits through reduced health care costs and productivity losses attributable to tobacco-related disease (30,31). Multiple economic evaluations show no negative effect on restaurant or bar revenue, and some report modest gains (31). Evidence from economic evaluations also shows that smoke-free laws do not harm restaurant or bar revenue and may yield downstream savings through reduced tobacco-related disease (30,31). At the same time, public support for smoke-free environments has grown steadily as awareness of SHS risks has increased (22). These findings show that strong public health protections are both feasible in entertainment-heavy regions and compatible with vibrant tourism and nightlife economies.

The parish-level trends observed in this study align with evidence from other US states and international settings, where comprehensive smoke-free ordinances have been associated with accelerated declines in tobacco-related morbidity and mortality rates, situating Louisiana within a broader and consistent evidence base of policy effectiveness (2,11,16,27). By leveraging natural variation in local policy adoption and analyzing 16 years of population-based cancer registry data from LTR, this study strengthens existing evidence by demonstrating differential cancer trends between parishes with comprehensive ordinances and those remaining under partial statewide protections. The use of Joinpoint regression enabled formal testing of differences in incidence slopes, enhancing inference within an ecological framework. The observed declines were notable in their magnitude, consistency, and timing, and no alternative local interventions were identified that would selectively influence Orleans and Ouachita parishes, supporting an association between comprehensive smoke-free policies and reduced cancer burden. Although this analysis focused on cancer outcomes, the benefits of comprehensive smoke-free policies are likely to continue accruing for other tobacco-related conditions, including cardiovascular disease and stroke, which contribute substantially to population-level illness and death (27,28).

Our study has limitations. Although race and ethnicity were incorporated through stratified analyses, other sociodemographic factors, including rurality, socioeconomic status, health care access, and occupational characteristics, could not be directly modeled using registry data and were therefore considered only in interpretation. The ecological study design does not permit causal inference at the individual level and does not allow adjustment for individual smoking behaviors. Statewide tobacco control efforts, including taxation, quitline promotion, and broader shifts in social norms, may also have contributed to observed trends (12,23). Given the long latency of many tobacco-related cancers, the full effects of more recent ordinances, particularly in Ouachita Parish, may not yet be observable (14,16,26). In addition, disruptions associated with the COVID-19 pandemic beginning in 2020 may have influenced diagnosis and incidence patterns (32). Although no contemporaneous local interventions or major demographic shifts unique to Orleans or Ouachita parishes were identified, unmeasured contextual factors cannot be excluded. Future studies using individual-level data, smoking prevalence trends, and occupational exposure measures would help clarify underlying mechanisms and more directly assess the contribution of policy implementation to observed cancer declines.

Comprehensive smoke-free ordinances were associated with significantly faster declines in lung cancer incidence compared with parishes covered only by Louisiana’s partial statewide policy, with Orleans Parish also demonstrating reductions across a broader group of tobacco-related cancers. Although unmeasured confounding remains possible, the pattern in Louisiana suggests that broader comprehensive smoke-free coverage may contribute to lower tobacco-related cancer burden and stronger protections for workers and residents over time. Expanding comprehensive smoke-free coverage statewide could accelerate declines in tobacco-related cancer prevalence, strengthen workforce protections, and help reduce persistent inequities in exposure and cancer outcomes. Louisiana’s experience emphasizes the effectiveness of comprehensive smoke-free policy as a structural cancer prevention strategy that is feasible in both urban and hospitality-driven environments.
